# In Vivo Comparison of Resin-Modified and Pure Calcium-Silicate Cements for Direct Pulp Capping

**DOI:** 10.3390/app151910639

**Published:** 2025-10-01

**Authors:** Fatma Fenesha, Aonjittra Phanrungsuwan, Brian L. Foster, Anibal Diogenes, Sarah B. Peters

**Affiliations:** 1 Division of Biosciences, College of Dentistry, The Ohio State University, Columbus, OH 43210, USA; 2 Department of Endodontics, School of Dentistry, University of Texas at San Antonio, San Antonio, TX 78229-3900, USA

**Keywords:** direct pulp capping, reparative dentin, calcium silicate, microleakage

## Abstract

**Introduction:**

Direct pulp capping (DPC) aims to preserve the vitality of the dental pulp by placing a protective biocompatible material over the exposed pulp tissue to facilitate healing. There are several calcium-silicate materials that have been designed to promote mineralization and the regulation of inflammation. These have strong potential for the repair and regeneration of dental pulp. Among them, Biodentine (BD) and EndoSequence RRM Putty (ES) have been found to promote in vitro and in vivo mineralization while minimizing some of the limitations of the first-generation calcium-silicate-based materials. Theracal-LC (TLC), a light-cured, resin-modified calcium-silicate material, is a newer product with potential to improve the clinical outcomes of DPC, but existing studies have reported conflicting findings regarding its biocompatibility and ability to support pulpal healing in direct contact with the pulp. A comprehensive assessment of the biocompatibility and pulpal protection provided by these three capping materials has not yet been performed.

**Aim:**

We aimed to quantify the inflammatory response, dentin bridge formation, and material adaptation following DPC using three calcium-silicate materials: ES, BD, and TLC.

**Materials and Methods:**

DPC was performed on the maxillary first molar of C57BL/6 female mice. Maxilla were collected and processed at 1 and 21 days post-DPC. The early inflammatory response was measured 24 h post-procedure using confocal imaging of anti-Lys6G6C, which indicates the extent of neutrophil and monocyte infiltration. Reparative mineralized bridge formation was assessed at 21 days post-procedure using high-resolution micro-computed tomography (micro-CT) and histology. Lastly, the homogeneity of the capping materials was evaluated by quantifying voids in calcium-silicate restorations using micro-CT.

**Results:**

DPC using TLC induced less infiltration of Lys6G6C^+^ cells at 24 h than BD or ES. BD promoted higher volumes of tertiary dentin than TLC, but TLC and ES showed no significant differences in volume. No differences were observed in material adaptation and void spaces among the three capping materials.

**Conclusions:**

All three materials under investigation supported pulp healing and maintained marginal integrity. However, TLC induced a lower inflammatory response on day 1 and induced similar levels of tertiary dentin to ES. These observations challenge the common perception that resin-based capping materials are not suitable for direct pulp capping. Our findings underscore the need to balance biological responses with physical properties when selecting pulp capping materials to improve long-term clinical success.

## 1. Introduction

Dental caries (tooth decay) is one of the most common chronic diseases worldwide [1,2], affecting people of all ages with a prevalence of 46.2% in primary teeth and 53.8% in permanent teeth [1–3]. The dental pulp is the innermost part of the tooth, a soft connective tissue that consists of nerves, blood vessels, and a wide array of cells. It plays a critical role in tooth formation, sensation, nutrition, and repair, and it ensures the long-term health of the tooth [4]. When the pulp is healthy, it keeps the tooth alive and functional. If the pulp becomes damaged or infected due to deep caries, trauma, or other factors, it can lead to pulp inflammation or pulpitis. Management of deep caries lesions, in which the infection extends close to or into the pulp, depends on the stage of inflammation (reversible or irreversible) and requires careful planning to preserve tooth vitality and prevent the recurrence of the infection [5–8]. Currently, the most widely used treatment modality for compromised pulp in adults is root canal therapy (RCT), which involves completely removing and replacing the dental pulp with a root canal sealing material. While this method has proven effective in saving the mineralized portion of the tooth, it does come with certain limitations, such as a high cost and greater likelihood for procedural accidents. Furthermore, root-filled teeth are at risk of secondary infections [9,10] that can progress to systemic problems due to the removal of the defensive and formative responses of the vital pulp. Due to the growing recognition of the crucial function of dental pulp, modern dentistry is increasingly focusing on preserving the vitality of this tissue, reflecting a shift from conventional RCT to less invasive dental procedures such as vital pulp therapy (VPT) [11–13].

The American Association of Endodontists defines VPT as “a treatment that preserves and maintains pulp tissue that has been compromised by trauma, caries or restorative procedures” [14]. In this sense, VPT can involve directly capping the dental pulp (DPC) or indirectly capping the dental pulp (IPC), and full or partial pulpotomy [7,15]. The success of VPT depends not only on the precise diagnosis and stage of inflammation, but also on the selection of the pulp capping agent [16,17]. The ideal pulp capping material should facilitate the formation of reparative dentin and the preservation of vital dental pulp [18]. Several pulp capping agents have been introduced into the market (Table 1), offering distinct advantages, disadvantages, and success rates. While some materials, like calcium hydroxide (CH), are widely recognized for their affordability and long history of use [19,20], they lack long-term durability and the ability to adhere to the dentin surface. In addition, CH has been shown to result in tunnel defects in the reparative mineralized bridge that forms [21–23], potentially leading to microleakage and bacterial invasion into the dental pulp [24].

To overcome the drawbacks of CH, calcium-silicate cement-based materials were introduced. These include Mineral Trioxide Aggregate (MTA) and, later, new-generation bioceramic materials. MTA was introduced as a therapeutic bioactive material that quickly gained popularity due to its biocompatibility, sealing ability, and excellent clinical success rates [25–27]. However, MTA has several limitations, including a high cost, a long setting time, tooth discoloration, and handling challenges. The newer generation of endodontic materials introduced some enhanced derivatives of calcium-silicate capping agents. Biodentine (Septodont, Saint-Maur-des-Fosses, France) is one MTA derivative that was developed as a dentin substitute [28–30]. The success rates of DPC with Biodentine were comparable to MTA in both short and long-term follow-up visits up to 24 months [19,25,31–33], but Biodentine exhibited better handling properties and a quicker setting time than MTA [34]. However, both MTA and Biodentine are supplied in powder/liquid form, which requires a separate mixing step before application, potentially leading to inconsistent chemical and mechanical capping properties due to variabilities in preparation by clinical staff [35,36].

To improve their handling and maneuverability in clinical settings, ready-to-use capping materials were developed, such as Theracal-LC (Bisco, Scaunburg, IL, USA), and premixed bioceramics, such as iRoot BP Plus, iRoot SP, iRoot FS (Innovative Bioceramics, Vancouver, BC, Canada), TotalFill RRM putty and paste (FKG, La-Chaux-de-Fonds, Switzerland), and EndoSequence RRM Putty (Brasseler, Savannah, GA, USA). These premixed bioceramics provide alkalinizing activity by releasing biointeractive ions (Ca+ and OH^−^) that induce apatite-like structure formation [37–39] to enhance dentin repair. Additional advantages have been reported, including a more uniform consistency, minimum technique sensitivity [40], antibacterial properties [41], and versatile applications in various endodontic procedures [42]. Despite their biological advantages, pure bioceramic calcium-silicate materials show limited bonding to overlying resin restorations due to their water-based chemistry, highlighting the need for improved adhesive strategies [33]. To address this limitation, Theracal-LC was developed as a hybrid light-cured resin-modified calcium-silicate material. The addition of the resin monomer provides improved physical properties and bonding, a shorter setting time, and better user-friendliness. However, there is concern regarding the potential cytotoxic effects of this material due to possible residual unpolymerized resin monomer in contact with the dental pulp tissue [43,44]. An extensive review by Arandi and Rabi [45] on Theracal-LC highlighted the lack of robust evidence supporting its use as a direct pulp capping material, emphasizing the need for further in vitro and in vivo studies.

These newer materials have generally been tested as endodontic sealers and root canal obturation agents, and few studies have been conducted to evaluate the use of these materials as pulp capping agents [38–40,46,47]. In practice, DPC agents are often applied in direct contact with the pulpal tissue, which can be strongly inflamed in cases with deep carious lesions or severe injuries. Therefore, gaining a deeper understanding of the immune responses elicited by these biomaterials is crucial to accurately assess their therapeutic potential. Most preclinical and clinical studies of calcium-silicate-based capping agents have focused primarily on reparative dentin formation as the main outcome [27,31,38,48,49], which relies on two-dimensional radiographs that lack the resolution to detect thin or early dentin bridge formation (<0.5 mm) [50]. Most material outcomes, including material adaptation, have only been investigated in in vitro studies [51–53], which often do not reflect tissue responses, particularly lacking information about the complexities of mastication and the dynamic microbiome. These limitations in assessment methods emphasize the need for more thorough in vivo analyses that include both the biological response and assessments of the material’s performance under clinical conditions.

The use of in vivo preclinical models allows for a multidimensional assessment by capturing the three-dimensional features of the capping materials and provides information about the dentin bridge formation based on cell- and tissue-level analyses. The aim of this study was to evaluate and compare the material and pulpal responses for three calcium-silicate capping materials: Biodentine (BD), EndoSequence RRM Putty (ES), and Theracal-LC (TLC). We assessed the immediate immune response (via immunofluorescence labeling), reparative dentin formation (via micro-CT and histology), and material homogeneity and adaptation (via void analysis using micro-CT). We hypothesized that resin-modified calcium-silicate materials, such as Theracal LC, would exhibit superior mechanical performance, while maintaining comparable pulp healing and biocompatibility to pure calcium-silicate materials like Biodentine and EndoSequence RRM Putty. We believe that this comparison of materials head-to-head in a controlled mouse model offers in-depth evidence to better inform material selection and future translational research in regenerative endodontics. This report also provides the details of how to perform this procedure in mice for future investigations into the molecular signals regulating reparative dentinogenesis.

## 2. Materials and Methods

This study incorporated the Preferred Reporting Items for Animal Studies in Endodontology (PRIASE) 2021 guidelines [79,80].

### 2.1. Animals

All mouse experiments were approved by the Institutional Animal Care and Use Committee (Protocol #2020A00000023-R1). Three-month-old wild-type (WT) C57BL/6 female mice weighing from 17 to 22 g were used. To ensure ethical and responsible conduct of animal research, the number of mice in this study was minimized in accordance with the ARRIVE 2.0 guidelines [81]. A sample size calculation using G*power software 3.1.9.6 (University of Düsseldorf, Düsseldorf, Germany) was carried out with the statistical power (1-β) set to 80%, the significance level set to α= 0.05, and a standard deviation of 5 for a sample size of 2 or 3 animals/group/assay to allow for detection of a 15% difference as a statistically significant difference. Hence, a total of 28 mice were used (12 for assessing the inflammatory response and 16 for measuring tertiary dentin formation and adaptation). The mice were maintained by qualified caretakers in a sterile setting with controlled humidity, temperature, and lighting.

### 2.2. Direct Pulp Capping Procedure

**Figure 1.**
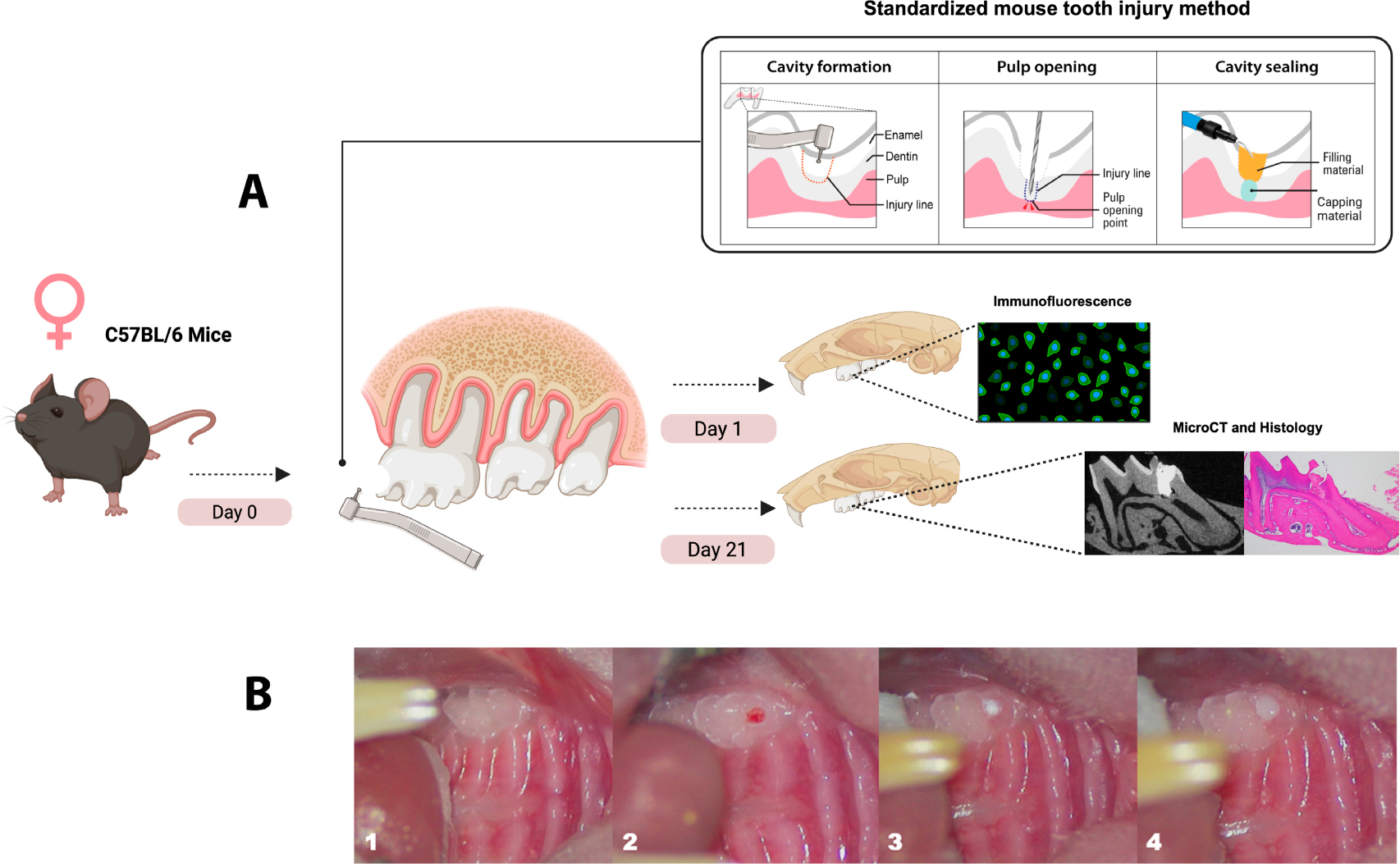
Experimental design and standardized pulp capping procedure. (**A**) Schematic representation of the experimental workflow and pulp capping technique using a standardized mouse tooth injury method. Samples of maxillae were collected at two time points: day 1 for immunofluorescence labeling of immune cells and day 21 for micro-CT and histological analysis. (**B**) Sequential images of the pulp capping procedure. (1) Unprepared maxillary first molar in a mouse. (2) Cavity preparation using a 0.3 mm round burr, followed by pulp exposure with a #15K endodontic file. (3) Application of the pulp capping material over the exposed pulp tissue. (4) Placement of a glass ionomer restoration to seal the cavity. Standardized mouse tooth injury illustration adopted from Yang et al. [82]. Illustration created with Biorender 2024.

The mice were randomly divided into 4 experimental groups (*n* = 3–4 mice/group) (Cavit-G (CG), G (3M ESPE, St. Paul, MN, USA) with a collagen sponge (Zimmer Plug^®^ and Optimax 3D^®^, Zimmer Biomet, Palm Beach Gardens, FL, USA), TLC, Theracal-LC (Bisco, Schaumburg, IL, USA), ES, EndoSequence RRM Putty (Brasseler, Savannah, GA, USA), and BD, Biodentine (Septodont, Saint-Maur-des-Fossés, France)). The mice were anesthetized with isoflurane and weighed before the experiment. We used a standardized mouse tooth injury model as described by Yang et al. [82] and shown in Figure 1A. A type-II or intermediate injury was performed involving the deep dentin with partial loss of pulpal tissue stimulating the formation of reparative dentin as part of the healing response [83]. The maxillary first molar was disinfected with 70% ethanol, and a cavity was drilled without exposure using a 0.3 mm round carbide burr (Komet, Fort Mill, SC, USA) on the mesial surface of both maxillary first molars with constant cooling. The mesial horn of the dental pulp was exposed using a #15 K endodontic file (Dentsply, Switzerland) with pinpoint bleeding confirming the exposure, after which hemostasis was achieved using a sterile paper point. The exposure was capped with one of the four capping materials (CG, ES, BD, or TLC), and then finally sealed with glass ionomer cement (Fuji IX, GC) as per the manufacturer’s instructions (Figure 1B). Each capping material was applied in a single layer and allowed to set according to the manufacturer’s instructions (Table 2). Excess material was removed, and the tooth and gingival tissues were cleaned with a cotton tip soaked in sterile distilled water. The mice were returned to their cages with no food for 30 min and then supplied with soft diet gel (W.F. Fisher and Son, Somerville, NJ, USA) for 24 h to minimize discomfort during mastication. The mice were then maintained on standard chow and monitored for changes in eating behaviors. Weights were recorded on day 0 and at 21 days post-capping.

### 2.3. Immunofluorescence Labeling

The mice were sacrificed at 24 h post-capping using intracardiac perfusion of 4% paraformaldehyde buffered with 0.5 M PB. Hemimaxillae were fixed in 4% paraformaldehyde for 2 h at room temperature and decalcified in poly-Nocal (Polysciences, Warrington, PA, USA) for 5–7 days. Half maxillae were then dehydrated with 30% sucrose at 4 °C and then cryopreserved. The total neutrophil and monocyte populations were labeled with an anti-Lys6G6C antibody (rat ab2557, Abcam 1:100) on day 1 post-capping. Cryosections (12 μm) were washed with phosphate-buffered saline containing Tween (PBST) and permeabilized with 1% Triton permeabilization blocking solution for 1 h, followed by incubation with anti-Lys6G6C primary antibody (rat Anti-Neutrophil, ab2557, Abcam 1:100) and then with Alexa Fluor secondary antibodies (A48269, Invitrogen, 1:500) overnight at 4 °C. The negative control slides stayed in block solutions and were not incubated with primary antibodies. DAPI was used to stain the nuclei (Invitrogen, 1:1000), then the stained slides were mounted and imaged using confocal microscopy (Olympus FV 3000 Confocal system, EVIDENT, Waltham, MA, USA) around the exposed mesial pulp horn and a similar region in the control non-injured maxillary molar. The images were converted to maximum projection for analysis using ImageJ 1.54h (National Institute of Health, Bethesda, MD, USA). Quantitative examination was carried out by measuring the integrated pixel density of Lys6G6C fluorescence within the same ROI using Image J. The ROIs were manually defined to include only the pulp tissue located directly beneath the pulp capping material. A fixed threshold was applied uniformly across all images to exclude background fluorescence. All sections were imaged using the same acquisition parameters and microscope settings to ensure consistency.

### 2.4. Histological Examination

The mice were sacrificed at 21 days post-capping via the intracardiac perfusion of 4% paraformaldehyde buffered with 0.5 M PB. Hemimaxillae were fixed in 4% paraformaldehyde for 2 h at room temperature and decalcified in poly-Nocal (Polysciences) for 5–7 days. Tissues were then dehydrated and embedded in paraffin and sectioned at 6 μm thickness along the midsagittal axis. The sections were stained using hematoxylin and eosin (H&E) or Masson’s trichrome stain according to standard protocols.

### 2.5. Micro-Computed Tomography (μCT)

Fixed hemimaxillae were scanned in 70% ethanol in a micro-CT scanner (Scanco Medical, Brüttisellen, Switzerland) set at 70 Kvp with a 6 μm voxel dimension, a 0.5 mm Al filter, and a 1200 ms integration time. Hydroxyapatite (HA) phantoms with defined density (0, 100, 200, 400, and 800 mg HA/cm^3^) were scanned at the same parameters and used to generate a standard linear curve to calculate the density unit of each sample, as mentioned in our previous article [84]. Reconstructed DICOM files were analyzed with AnalyzePro 15.0 (AnalyzeDirect). The first maxillary molar and associated alveolar bones were segmented as regions of interest (ROIs). The ROI was outlined as the area between 240 μm mesial from the most mesial point of the mesial maxillary first molar root and 240 μm distal from the most distal point of the distal maxillary molar root. Reparative dentin was manually segmented at 240–1600 mg HA/cm^3^. The periodontal ligament (PDL) space of the mesial maxillary molar was manually segmented using the apical third of the total mesial root length.

The adaptation of the four capping materials was assessed by measuring the void percentage in the capping materials. The total volume of each capping material and the internal void volume were quantified using high-resolution micro-CT. The capping material was outlined, and the voids were manually segmented at 2300–2811 mg HA/cm^3^. This range was selected to effectively exclude enamel from our analysis, as enamel possesses a significantly higher mineral density compared with dentin and other dental tissues. Segmentation was performed based on grayscale thresholding to differentiate between the capping material and voids. All measurements were conducted using consistent threshold values and image analysis parameters to ensure consistency across samples. The void percentage of each capping material was calculated using the following equation: Void percentage = (void volume/total void + capping volume) × 100.

### 2.6. Statistical Analysis

Data are presented as the mean ± standard deviation in the graphs. The normality of data was assessed using the Shapiro–Wilk test. Data were evaluated with a one-way analysis of variance (ANOVA) and Tukey’s multiple comparisons test to compare between the capping materials, where *p* ≤ 0.05 was considered statistically significant. Data analysis was performed using statistical software (Prism 10.4.1; GraphPad Software). The sample size calculation was based on previous studies evaluating comparable outcomes [31,38]. A total of 16 mice (3–4 mice per group), each contributing bilateral molars, were deemed sufficient to detect statistically significant differences.

## 3. Results

No adverse events occurred, nor were any mice lost during the experiments. All mice had maintained their coronal restoration up to the time of sacrifice.

### 3.1. Theracal-LC Led to Reduced Monocyte and Neutrophil Recruitment Compared with EndoSequence RRM Putty and Biodentine at 24 H

**Figure 2.**
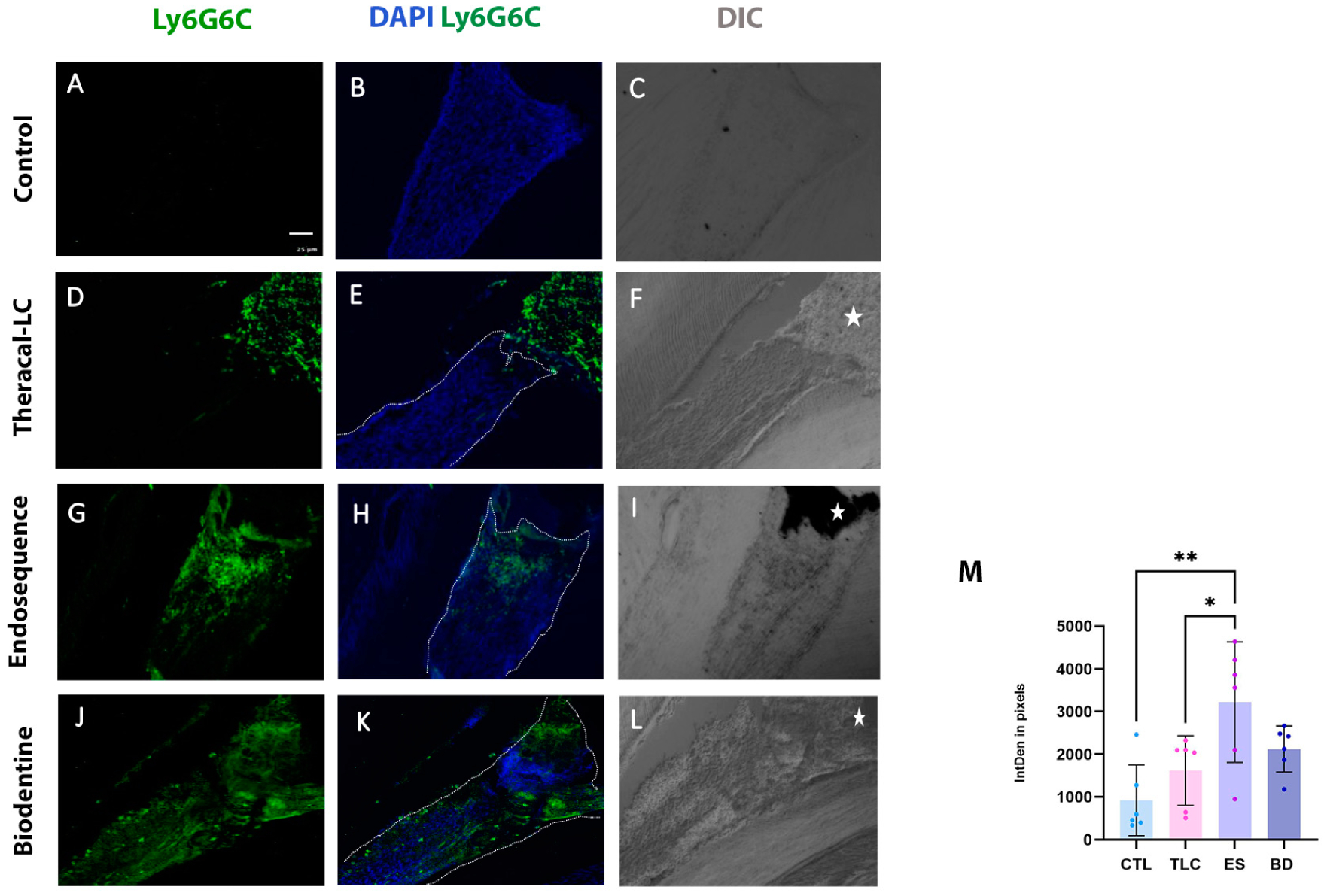
Immunofluorescence labeling of neutrophils and monocytes at 24 h. Representative confocal images showing the Ly6G6C^+^ cells (green), labeling neutrophils and monocytes, across all capping material groups at 24 h (**D**–**L**). (**A**–**C**) Non-injured (CTL) dental pulp demonstrating no LY6G6C^+^ cells. DAPI is stained in blue. The dental pulp is outlined with a white dotted line in the merged images and the capping material is highlighted with a white asterisk in the Differential Interference Contrast (DIC) images. (**M**) Comparison of Ly6G6C^+^ expression (integrated pixel density) between the non-injured (CTL) group and the capping material groups (TLC, ES, and BD). The bars represent the mean percentage of Ly6G6C^+^ cells for each experimental group. Each dot represents a (sample/animal) within that group. * *p* < 0.05, ** *p* < 0.01 by one-way ANOVA. N = 3 mice (with two consecutive slides analyzed per animal). Scale bar: 25 μm in (**A**). CTL, non-injured control; TLC, Theracal-LC; ES, EndoSequence RRM Putty; BD, Biodentine.

As shown in Figure 2A–L, immunofluorescence labeling revealed increased early recruitment of Ly6C6G+ monocytes and neutrophils within 24 h of the direct pulp capping procedure. Both ES and BD exhibited more Ly6G6C^+^ cells than TLC, indicating a more pronounced initial immune response (*p* < 0.05) (Figure 2M). No significant differences were observed between ES and BD (*p* > 0.05). Notably, there was also no significant difference in immune cell recruitment between the TLC group and the controls (*p* > 0.05).

### 3.2. Mineralized Bridge Formation Induced by Calcium-Silicate-Based Pulp Capping Agents

**Figure 3.**
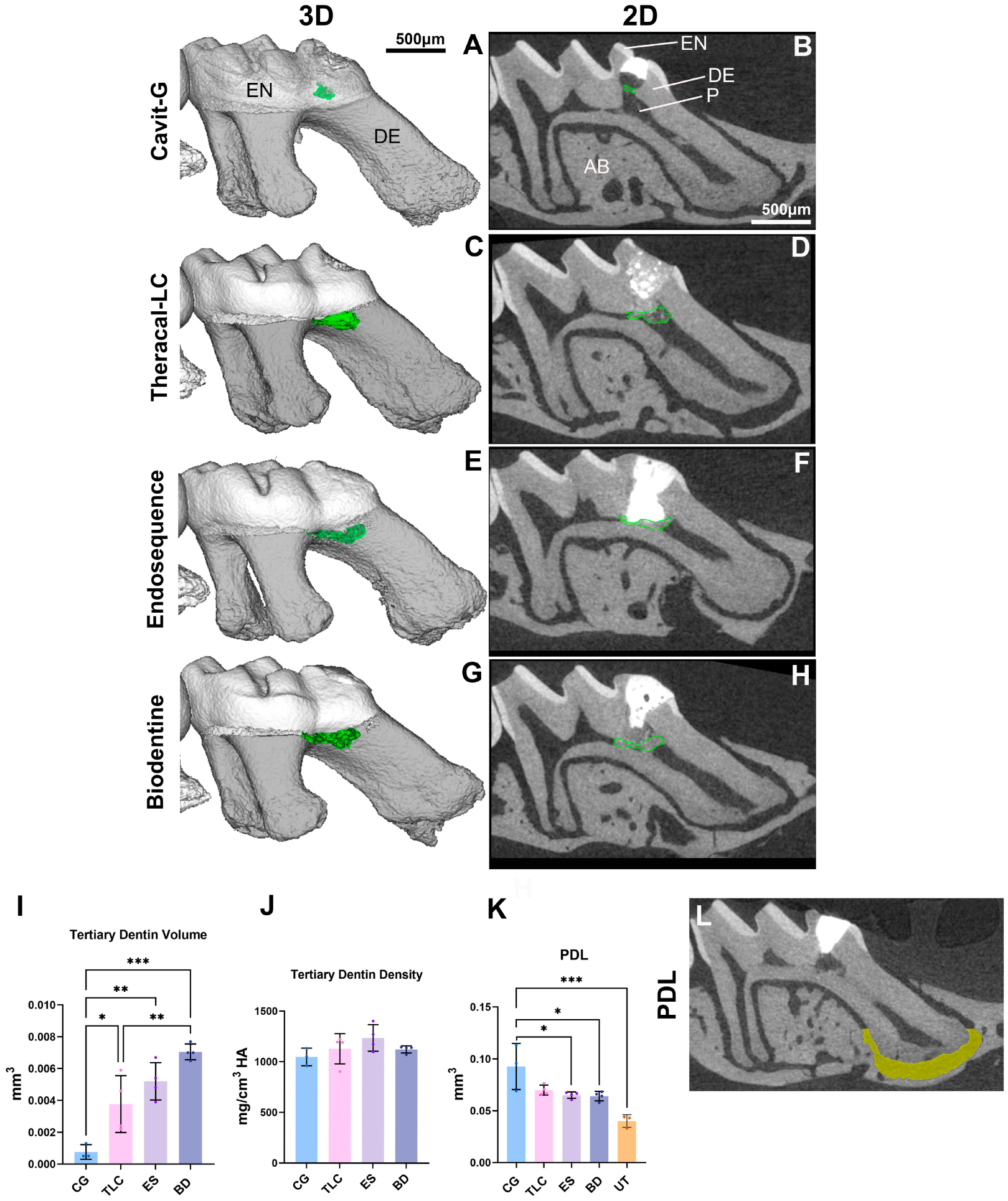
Micro-CT analysis of reparative dentin formation and PDL widening at 21 days post-capping. (**A**,**B**) Cavit-G group (negative control) displaying signs of random opacities along the root canal. (**C**–**H**) Representative 3D and 2D slices demonstrating the formation of reparative dentin (outlined in green), which appeared denser than for Cavit-G, and was localized beneath the pulp capping material. Quantification of the reparative dentin volume (**I**) and density (**J**) showing significant differences in pulp capping volume between and among each capping material (TLC, ES, BD, and CG). No significant differences were observed in the density among the four materials. The region of interest (ROI) used to assess PDL widening is represented in (**L**). The PDL was manually segmented at the apical 1/3 of the total mesial root length of the injured maxillary first molar. Quantification of the PDL widening presented in (**K**); significant differences were observed between the BD and CG, ES and CG, and UT (untreated) and CG groups. No significant differences were observed between any of the three capping materials (TLC, ES, and BD) and the UT group. Scalebars are 50 μm in (**B**). * *p* < 0.05, ** *p* < 0.01, *** *p* < 0.001 by one-way ANOVA. N = 4 mice. P, pulp; DE, dentin; EN, enamel; AB, alveolar bone; CG, Cavit-G; TLC, Theracal-LC; ES, EndoSequence RRM Putty; BD, Biodentine.

**Figure 4.**
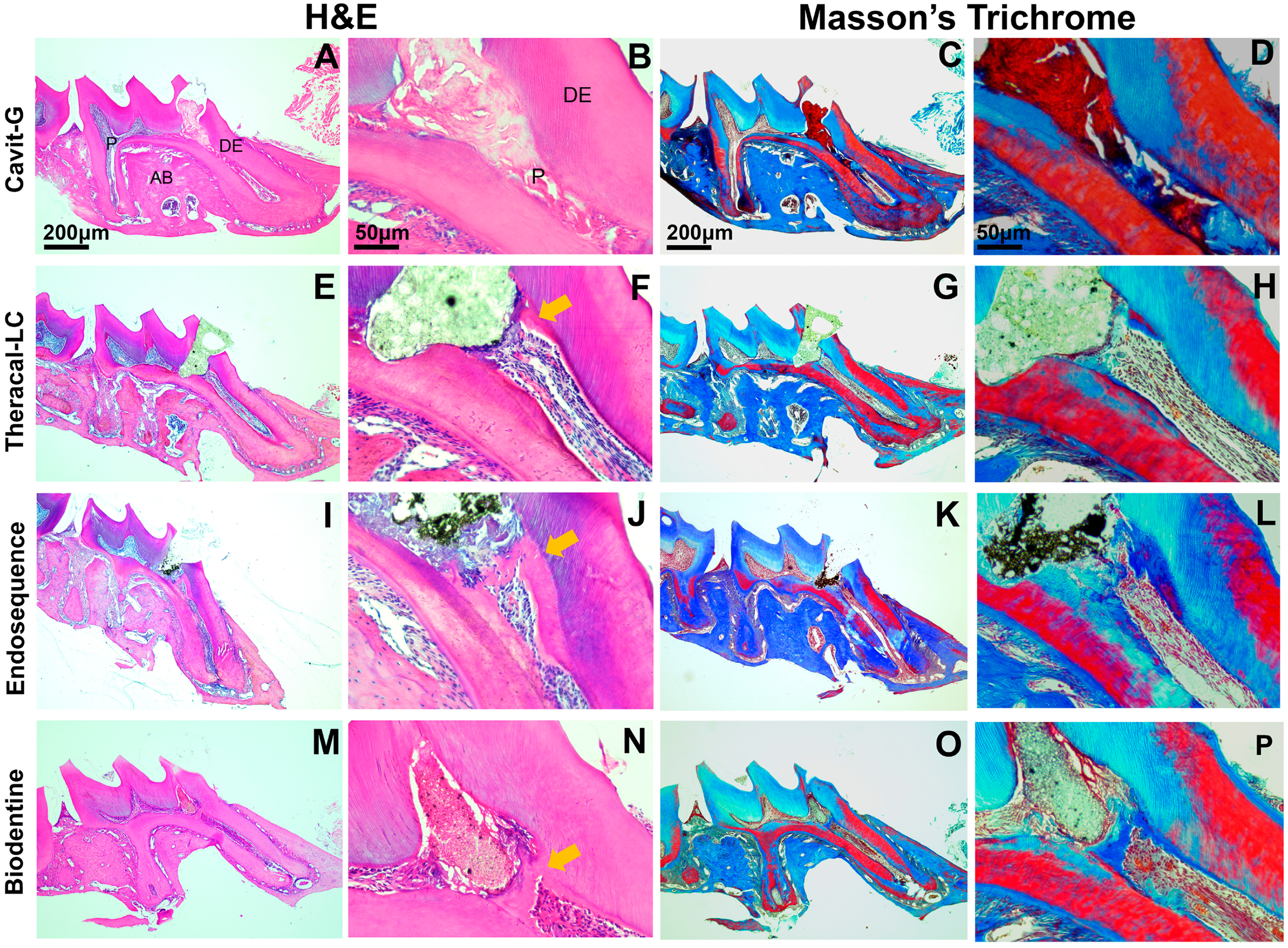
Histopathological evaluation on day 21 post-capping using hematoxylin–eosin and Masson trichrome staining. (**A**–**D**) The Cavit-G group (negative control) displayed no evidence of reparative dentin, the absence of odontoblasts and dental pulp cells, and extension of inflammation along the mesial root canal. (**E**–**H**) The Theracal-LC group, (**I**–**L**) EndoSequence RRM Putty group, and (**M**–**P**) Biodentine group all showed signs of repair. Images (**E**–**P**) demonstrate reparative dentin formation, indicated by the yellow arow in (**F**,**J**) and (**N**). Scalebars = 200 μm in (**A**) and (**C**) and =50 μm in (**B**) and (**D**). P, dental pulp; DE, dentin; AB, alveolar bone.

Micro-CT was employed to assess the formation of tertiary dentin and the morphology of the PDL space (Figure 3A–H,L). At 21 days, the CG group exhibited significantly less tertiary dentin formation compared with the other groups. Ectopic calcifications, occurring at sites distant from or non-adjacent to the injury (Figure 3A,I), and pulpal inflammation (Figure 3K–L and Figure 4A–D) were observed in the CG cohort. A complete dentin bridge was evident with no signs of tunnel defects with all three materials (ES, BD, and TLC) (Figure 4E–P). The tertiary dentin volume was lower in the TLC group compared with the BD-treated group. However, there was no significant difference in the dentin volume between the TLC and ES nor between the BD and ES groups (Figure 3I). No significant differences were detected in the density of tertiary dentin among experimental groups (Figure 3J). An analysis of the PDL space surrounding the apical one-third of the mesial root was conducted to evaluate the longer-term inflammatory response elicited by each capping material. The CG restorations led to a significantly enlarged PDL space relative to the untreated group. However, we did not find any significant differences in PDL widening between the untreated group and the TLC, ES, or BD group (Figure 3K,L).

### 3.3. All Calcium-Silicate-Based Pulp Capping Agents Showed Comparable Marginal Adaptation

**Figure 5.**
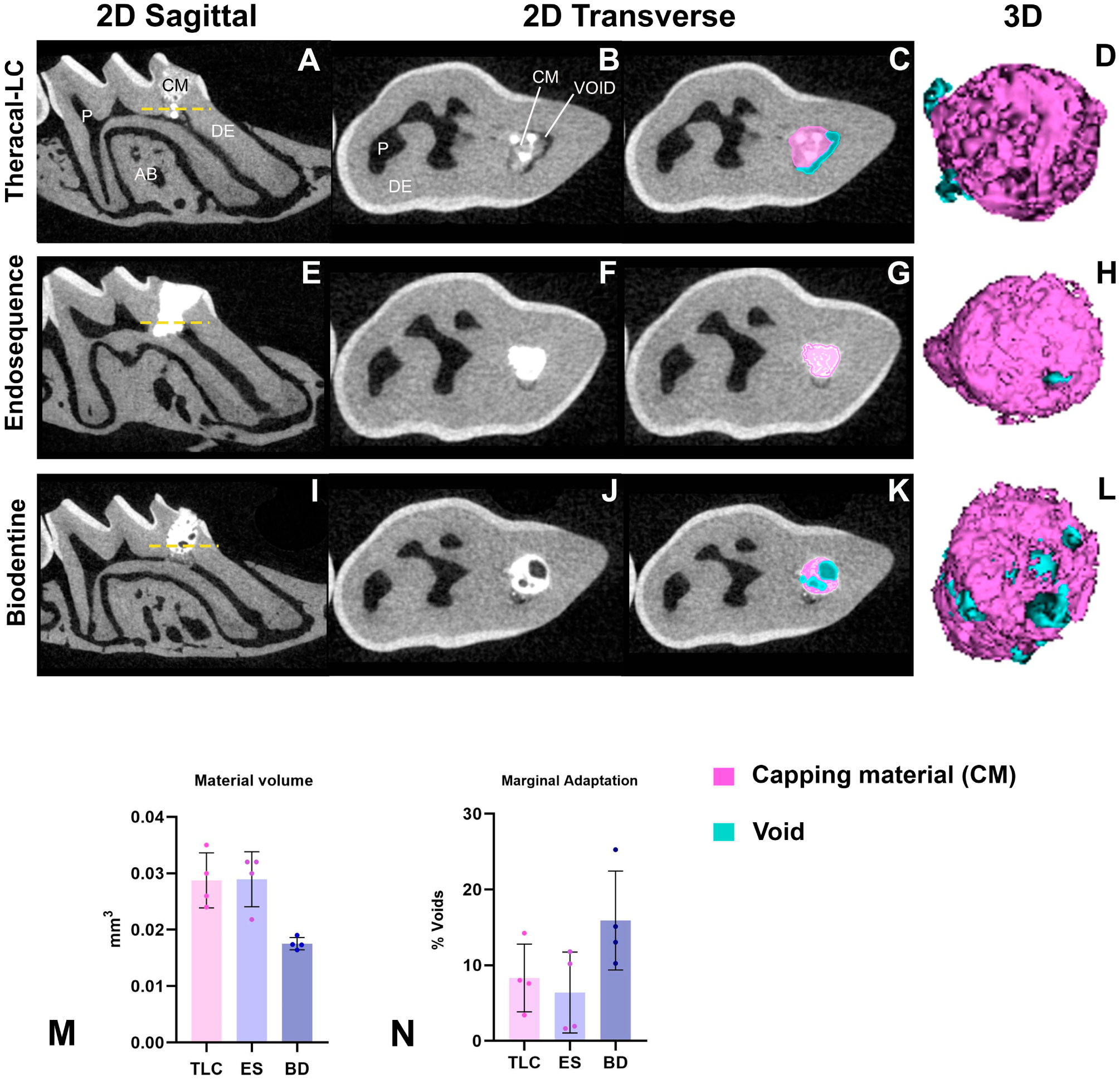
Micro-CT analysis of the marginal adaptation of the capping materials at 21 days post-capping. This illustration shows sagittal cross sections of the capped first maxillary molar presented in (**A**,**E**) and (**I**). Transaxial 2D slices sectioned at the dashed yellow line in (**B**–**L**) displaying the total capping and void areas in TLC, ES, and BD. Three-dimensional rendering of the three capping materials (purple) and associated voids (blue). Quantification of the capping material volume (**M**) and void % in (**N**) for the three capping materials (TLC, ES, and BD). Scalebars are 100 μm. By one-way ANOVA. N = 4 mice. P, pulp; DE, dentin; AB, alveolar bone; CM, capping material; TLC, Theracal-LC; ES, EndoSequence RRM Putty; BD, Biodentine.

Direct pulp capping with all tested calcium-silicate-based materials (TLC, ES, and BD) demonstrated comparable homogeneity and adaptation to the pulp–dentin interface (Figure 5A–M). No significant differences were observed among the TLC, ES, and BD groups in terms of either the void percentage or total capping material volume. Figure 5 presents representative 2D slices and 3D segmentations illustrating the total capping and void volumes.

## 4. Discussion

There has been a growing interest in VPT approaches to preserve and restore the dental pulp vitality and its important physiological functions [85–89]. Traditionally, the focus of VPT was maintaining the vitality of the radicular dental pulp after minor injuries or caries exposure, specifically for primary teeth and immature permanent teeth with deep caries, to allow for continued root formation and apex closure [90]. Today, VPT has evolved to have a broader scope, no longer restricted to immature teeth, with advances in biomaterials and minimally invasive techniques making VPT a viable option for mature permanent teeth [5]. Despite the potential of VPTs to enhance pulp healing in older adults, the reported success rates still vary widely (from 42.9% to 81.5%) and are generally lower compared with full pulpectomy [23,91]. However, the variability is largely due to the lack of comprehensive studies using specific clinical outcomes. Therefore, the present study was undertaken to provide an in-depth investigation of the underlying factors that may contribute to these wide variations in success by directly comparing the three main materials when they were used for the same model injury.

Several additional challenges remain that hinder the widespread clinical adoption and success of VPTs, necessitating further research. Currently, there is no consensus among clinicians on the management of deep carious lesions, largely due to the lack of comparative studies investigating the outcomes of VPTs and their long-term success [92]. This also reflects the practical difficulty of designing parallel clinical trials, given the ethical considerations of managing pain and pulpal infections in human patients. An accurate diagnosis of the stage of inflammation also remains a challenge, as the absence of a histological reference standard limits reliability; studies have demonstrated poor correlation between clinical symptoms and histological findings [93,94]. Finally, the selection of pulp capping agents capable of promoting high-quality dentin formation is a critical determinant of VPT success. MTA has largely replaced calcium hydroxide as the gold standard for direct pulp capping due to its superior sealing ability, biocompatibility, and long-term clinical outcomes [26]. Newer calcium-silicate-based cements were introduced to address several drawbacks associated with traditional MTA, including the long setting time, difficult handling properties, discoloration, and relatively high cost. Materials such as Biodentine, EndoSequence RRM Putty, and Theracal-LC were developed to improve clinical usability through faster setting times, premixed formulations, and enhanced mechanical properties, while retaining the biological benefits of MTA. Despite promising in vitro and clinical data, direct comparisons of in vivo pulpal responses between these newer materials remain limited. In the present study, we focused on comparing three commercially available and accessible calcium-silicate pulp capping agents to help guide evidence-based material selection and translate the findings into improved clinical outcomes. Our results reveal that Theracal-LC induced reparative dentin formation by day 21, demonstrating similar biocompatibility to Biodentine and EndoSequence RRM Putty, and confirming that it has the capacity to support pulp healing. The treatment with Cavit-G, a non-bioactive material, highlighted the biocompatibility of TLC, BD, and ES, as significant differences in tertiary dentin volume, ectopic calcifications, and PDL widening were observed between the Cavit-G-treated negative control and the three calcium-silicate materials. No significant differences in dentin density were detected among any of the groups. Our findings are in agreement with previous studies [22,31,44,49] evaluating calcium-silicate-based materials as pulp capping agents, further supporting their effectiveness in promoting reparative dentin formation and pulp healing.

A key factor affecting the success of VPT is the immune response induced by the materials in contact with the pulp. The dental pulp is dynamically regulated by sequential activation of the innate and adaptive immune responses to external stimuli [95,96]. Accordingly, as the pulp capping material provides a protective barrier over the dental pulp, it is expected to promote a shift from inflammation to repair, aiming to preserve the pulp vitality [92]. The first few hours after the material application represents a critical time where the innate immune response is rapidly activated and then transitions to tissue repair. This early stage is characterized by the rapid recruitment of immune cells to the site of injury, which plays a crucial role in clearing inflammation and prompting tissue repair [97,98]. Neutrophils play an essential role in the acute inflammatory response as they act as the first line of defense against infective pathogens. However, neutrophils begin to undergo apoptosis after 24 h, helping to resolve the inflammatory response [97,99]. Additionally, this programmed cell death induces the migration of other immune cells from the bloodstream, such as monocytes, which also play a critical role in the early defense response as well as mediate the transition into the adaptive immune phase [97,100,101].

Defense and repair are tightly connected and regulated responses within the dentin–pulp complex. A low-grade inflammatory response is necessary to initiate tissue healing, and, in the context of reparative dentinogenesis, both neutrophils and monocytes are crucial for regulating early-stage inflammation [97,102]. In our study, we focused on evaluating the innate immune response on day 1 to assess early biological responses and to identify potential differences in the initial inflammatory response. While all three capping materials triggered an immune response at this early time point, Theracal-LC was associated with the mildest response, as indicated by the lowest number of infiltrating monocytes and neutrophils, as assessed by immunofluorescent labeling of Ly6G6C. This could be attributed to the low percentage of monomer components (5–10%) in this formula of resin-modified calcium-silicate cements. This is consistent with the manufacturer’s safety data sheet (2012), indicating that the material is 2-hydroxyethyl methacrylate (HEMA)-free and primarily composed of Portland cement, polyethylene glycol dimethacrylate, and barium zirconate. Additionally, this was supported by Nilsen and Einar, who found no evidence of Bisphenol A-glycidyl methacrylate (Bis-GMA) in their analysis of Theracal-LC [103]. Further, the inflammatory responses appear to be related to the materials used, as minimal inflammation was seen in the non-injured healthy control molars.

Another key factor for the success of VPT is achieving a well-adapted void-free seal to prevent microleakage. Inadequate adaption is a major cause of failure in endodontic procedures, as it allows for bacterial penetration, leading to recurrent inflammation, tooth decay, and compromised treatment outcomes [52,53]. A material that promotes dentin formation but fails to provide an effective marginal adaptation would not be clinically viable for long-term performance. In this study, we utilized high-resolution micro-CT to evaluate the homogeneity of different capping materials by measuring the void percentages. We did not find a significant difference in the marginal adaptation between the Biodentine, Theracal-LC, and EndoSequence RRM Putty. However, studies have reported higher bond strength and dentin adhesion with resin-modified pulp capping agents compared with other agents. In an in vitro study, Makkar et al. evaluated the adaptation of Biodentine and Theracal-LC against conventional MTA using confocal laser microscopy and Rhodamine B fluorescent dye. Both Biodentine and MTA showed comparable micropermeability at the material dentin interface. However, Theracal-LC exhibited significantly higher adaptation than the other two [104]. One possible explanation for the lower homogeneity of Biodentine is the variability introduced by its powder-to-liquid mixing process, which may lead to inconsistencies in material properties compared with ready-to-use materials. Additionally, achieving a strong bond between the pulp capping material and the final restoration is critical for the success of VPTs. Theracal-LC has been shown to provide superior bond strength to composite resin and resin-modified glass ionomer restorations compared with water-based calcium-silicate materials [105–108].

TheraCal-LC (TLC) combines the bioactivity of calcium silicates with the handling advantages of resin. Its hydrophilic nature permits ion exchange and calcium release, supporting reparative dentinogenesis. Unlike Biodentine and EndoSequence RRM Putty, which are well-studied and widely accepted for DPC, Thercal-LC’s biocompatibility and reparative potential in direct contact with pulp remain controversial. Our findings suggest that resin-based calcium-silicate materials such as Theracal-LC can be used safely in direct contact with the pulpal tissue, while providing a well-sealed protective barrier. In light of this, Theracal-LC is an attractive material of choice, due to its light-cured settings, which facilitate immediate placement of the final sealing material and allow for layering of restorative materials in deep cavities that could be completed in the same visit. This is particularly useful for pediatric patients and clinical situations where reduced chair time is needed. Future investigations into the long-term performance of Theracal-LC compared to non-resin-based bioceramics is recommended to best determine its efficacy in providing bonding, adhesion, and protection of the tooth organ as a whole.

This study offers several important strengths contributing to the growing body of evidence regarding the use of calcium-silicate-based materials as pulp capping agents. First, the study employed a standardized and reproducible pulp capping technique, ensuring uniform application of materials and minimizing procedural variability. All procedures were performed by a single trained investigator to ensure consistency and minimize inter-operator variability. Second, the combined use of micro-CT imaging and histological analysis for the quantification of early immune reactions and reparative dentin volume offers a more nuanced understanding of material performance than studies relying on a single outcome measure. Assessing the homogeneity of a pulp capping material is crucial to ensure its long-term success and durability. Lastly, comparing three widely used calcium-silicate materials head-to-head under identical experimental conditions helps determine whether the materials’ performance is suitable for real-world clinical performance, informing potential clinical applications.

This in vivo study was conducted to address the inconsistency and lack of standardization in previous VPT research, particularly the use of varying animal models. A standardized DPC mouse model was necessary to enable direct comparison. Both our work and that by previous investigators [82] has demonstrated the utility of this model. Nonetheless, this study has certain limitations. The first is the use of sound healthy molar teeth rather than a pulpitis model. While sterile exposed healthy pulp allows for controlled comparisons of reparative dentinogenesis and inflammation, this does not fully replicate the clinical environment where pulp capping is typically indicated. As such, the healing and inflammatory dynamics observed in this model may differ from those in a diseased state, potentially affecting the translational relevance of the findings. The objective of this study was to determine whether the materials could independently induce dentin bridge formation, without the confounding influence of microbial-driven inflammation. This allows us to establish a baseline understanding of their reparative potential and the capacity of materials to support repair in the absence of infection. Therefore, our conclusions are most applicable to contexts such as dental trauma or cases with reversible pulpitis, or where microbial challenge is minimal. It is important to note that the observation period of 21 days does not address dentin maturation, long-term sealing ability, or the risk of chronic inflammation and necrosis. In this study, we aimed to capture early healing events and the initial formation of reparative dentin to address the biocompatibility. In rodents, one month roughly equates to three human years [109]; thus, 21 days approximates nearly two years of reparative processes in human studies. Future studies with an extended time point (up to 56 days) are warranted to determine the long-term outcomes of each material. Finally, while micro-CT provides a valuable volumetric assessment of voids and material placement, it does not capture the microscopic interaction between the capping material and dentinal surfaces [110,111]. This includes adhesion at the dentin interface and penetration into dentinal tubules, which are critical for long-term sealing. Future studies should incorporate advanced techniques to better characterize capping material adaptation and bonding at the dentin interface and with the overlaying restoration.

Although calcium-silicate-based materials have shown promising results in preclinical and in vitro studies [19,25,31–33,37,38], translating these findings to clinical success in human teeth is not always straightforward. This is because studies have often been conducted using different animal models (e.g., rats, dogs, or mice), which each have unique pulp anatomies, healing capacities, and immune responses that differ significantly from human teeth [26,112]. The choice of model depends on the specific research goals, ethical considerations, and resources available. Rats have traditionally been the most widely used preclinical models in endodontic research due to their relatively large molar size, cost-effectiveness, and ease of handling [113]. However, mouse models allow for global and conditional deletion and/or overexpression of genes to investigate their role(s) in the pulp tissue response and healing. Our study has compared calcium-silicate cements that are widely used in VPTs in a well-established, standardized, and reproducible model in mice, enabling consistent assessment of pulp healing and material performance that could be used in future studies of specific parameters that might be manipulated to improve the clinical outcomes.

## 5. Conclusions

Theracal-LC induced a lower inflammatory response on day 1, while promoting reparative dentin formation and providing a reliable protective seal over the dental pulp at 21 days, similar to Biodentine and EndoSequence RRM Putty. These observations indicate the suitability and potential superiority of Theracal-LC as a direct pulp capping agent. Our findings underscore the importance of evaluating both biological and physical properties to inform the selection of pulp capping materials in clinical practice.

## Figures and Tables

**Table 1.** Summary of common pulp capping materials.

Material	Composition	Advantages	Disadvantages	Setting Type/Time
Dycal Pulpdent Pulpcal	Base: Zinc oxide, calcium phosphate, calcium tungstate Catalyst: Calcium hydroxide, zinc oxide, and titanium dioxide	Easy to work with, sets quickly, cost-effective [20]	Limited antibacterial activity over time [54] Poor cohesive strength [55] Greater solubility and marginal leakage [56,57] Tunnel defect in formed reparative dentin [21,22]	Self-curing 2–3 min
Cavit-G Zinogen	Zinc oxide eugenol	Reduces inflammation [58]	Releases eugenol, which even at low concentrations can be cytotoxic to dental pulp cells [59,60]	Self-curing 5–10 min setting, with curing continuing for 1–2 h
GIC Fuji	Glass ionomer/resin-modified glass ionomer	Excellent bacterial seal [61] Fluoride release [62,63] Bonds to both enamel and dentin Good biocompatibility [64]	Cytotoxic when in direct contact with cells, and does not lead to dentin bridge formation [41,65]	Self-curing 3–5 min
Mineral trioxide aggregate (MTA)	Tricalcium and dicalcium silicate, tricalcium aluminate, tricalcium and silicate oxides, and bismuth oxide	Good biocompatibility [7,27] More predictable hard tissue barrier formation than CH [66] Antibacterial properties [67] Osteoinductive [68]	Poor handling characteristics [69] Long setting time [70]	Self-curing 30 min–2 h
Biodentine	Powder: tricalcium silicate, calcium carbonate, and zirconium oxide Liquid: water and calcium chloride	Biocompatible [71] Bonds to deep moist dentin [32,72] Induces mineralized bridge formation [48] Faster setting time and good physiochemical properties [28]	Shorter working time and limited radiopacity [73] Difficulty in achieving the desired consistency Poor bonding with overlying restorations [74]	Self-curing 12 min
Theracal LC	Calcium silicate, barium sulphate, barium zirconate, fumed silica, and Bis-GMA and PEGDMA resin	Low solubility and good mechanical properties [75] Easy handling, convenient, and short setting time [76] Induced dentin bridge formation [77]	Long-term efficacy is limited [47] Higher cytotoxic effects and reduced cell viability [71] Reduced radiopacity	Light-cured 10 s
Premixed Bioceramics (EndoSequence BC, TotalFill BC RRM, iRoot BP Plus)	Calcium silicate, zirconium oxide, calcium phosphate, calcium sulphate, and fillers.	Superior handling, uniform capping consistency and convenient manipulation [78] Biocompatible [71] Ability to induce mineralization and odontoblast differentiation [37]	Limited long-term data [40] Tooth discoloration [42]	Self-curing 10–20 min

**Table 2.** Application method and setting time of each capping material.

Capping Material	Material Application	Setting Time
Biodentine (BD):	BD powder and liquid were mixed according to the manufacturer’s instructions to form a putty-like paste. The powder capsule was placed into the provided stand and 5 drops of liquid were added, after which the capsule was closed and placed into the 3M ESPE RotoMixer (3M ESPE, Solventum, Solventum, MN, USA) for 30 s at 4000 vibrations per minute. The mixed BD paste (<1 mm) was gently placed over the pulp with a micro-spatula and allowed to set before final restoration.	12 min
Theracal-LC (TLC):	After achieving gentle hemostasias with a sterile paper point, a thin layer not exceeding 1 mm of TLC was applied directly to the pulp exposure site using a micro-spatula. Polymerization of TLC was performed using a two-LED-based curing blue light for 20 s with a wavelength ranging from 420 to 490 nm and an output intensity of 1200 mW/cm^2^ (Essentials, The Dentistry Supply Company, Inc., Melville, NY, USA), after which the final restoration was placed over the TLC to seal the cavity.	20 s
EndoSequence RRM Putty (ES):	Leaving the dentin lightly moistened, 0.5–1 mm of ES putty was delivered with a sterile applicator and gently adapted to the pulp surface. As a moisture-setting material, it was allowed to harden before applying the final restoration.	10 min

## Data Availability

The original contributions presented in this study are included in the article. Further inquiries can be directed to the corresponding authors.
